# Leucémie aiguë lymphoblastique révélée par une atteinte du sinus caverneux chez un jeune homme

**DOI:** 10.11604/pamj.2014.19.98.5088

**Published:** 2014-09-26

**Authors:** Badreeddine Alami, Mustapha Maaroufi

**Affiliations:** 1 Service de Radiologie, CHU Hassan II, Fès, Maroc

**Keywords:** Leucémie aiguë, sinus caverneux, dissémination hématogène, acute leukemia, cavernous sinus, hematogenous spread

## Image en medicine

L'atteinte du sinus caverneux au cours des leucémies aigues est rare et peut se faire par extension directe ou via une dissémination hématogène. L'aspect en imagerie n'est pas pathognomonique mais reste évocateur: le scanner montre une infiltration tissulaire du sinus caverneux, l'IRM est plus performante montrant un hypo signal T2 de cette infiltration, avec restriction de la diffusion et prise de contraste après injection du Gadolinium. Nous rapportons le cas d'un jeune homme, âgé de 22 ans, admis aux urgences dans un tableau d'HTIC évoluant depuis 10 jours avec diplopie horizontale. Une TDM cérébrale a objectivé un aspect élargi des 2 sinus caverneux qui sont spontanément hyperdense rehaussés de façon modérée après injection du produit de contraste. La NFS a montré une thrombopénie avec un taux de plaquettes à 33000/mm^3^, une hyperleucocytose à 16700/mm^3^, le taux d'hémoglobine était à 13.7g /l. Nous avons complété le bilan d'imagerie par une IRM cérébrale qui a montréune infiltration tissulairedes 2 sinus caverneux se présentant en hypo signal T2, avec restriction de la diffusion et prise de contraste homogène après injection du gadolinium. Une atteinte lymphomateuse a été évoquée sur cet aspect. 2 jours après le patient a présenté des hématomes à chaque point d'injection. Une NFS avec frottis ont montré un taux de plaquettes à 21 000/mm^3^ et des blastes à 25%, ensuite un myélogramme a été réalisé revenant en faveur d'une leucémie aigue lymphoblastique. L’évolution a été fatale par hémorragie digestive.

**Figure 1:**
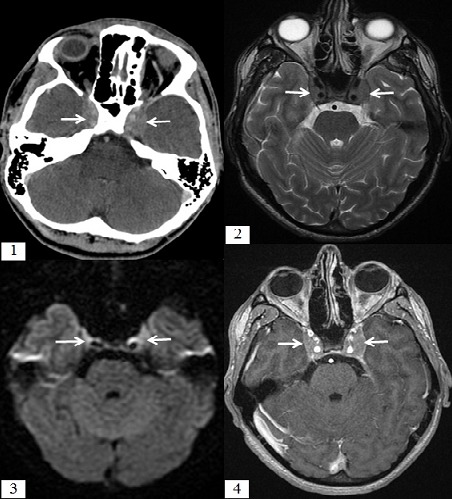
(1): TDM cérébrale en Coupe axiale, après injection du produit de contraste montrant une infiltration tissulaire des deux sinus caverneux (flèche); (2): IRM cérébrale, coupe axiale en séquence T2 objectivant un aspect élargi des 2 sinus caverneux en rapport avec une infiltration tissulaire se présentant en hyposignal T2 (flèche); (3): IRM cérébrale, coupe axiale en séquence de diffusion montrant un hypersignal (restriction de la diffusion) au sein des 2 sinus caverneux (flèche); (4): IRM cérébrale, coupe axiale en séquence 3D T1 après injection du gadolinium objectivant un rehaussement homogène de l'infiltration tissulaire des deux sinus caverneux qui sont perméables (flèche)

